# Treatment of glioma patients with ketogenic diets: report of two cases treated with an IRB-approved energy-restricted ketogenic diet protocol and review of the literature

**DOI:** 10.1186/s40170-015-0129-1

**Published:** 2015-03-25

**Authors:** Kenneth Schwartz, Howard T Chang, Michele Nikolai, Joseph Pernicone, Sherman Rhee, Karl Olson, Peter C Kurniali, Norman G Hord, Mary Noel

**Affiliations:** Department of Internal Medicine, Michigan State University, East Lansing, MI 48824 USA; Department of Pathology, Sparrow Hospital, Lansing, MI 48912 USA; Department of Neurology and Ophthalmology, Michigan State University, East Lansing, MI 48824 USA; Department of Family Medicine, Michigan State University, East Lansing, MI 48824 USA; Department of Radiology, College of Human Medicine, Michigan State University, East Lansing, MI 48824 USA; Department of Clinical Nutrition Services, Sparrow Hospital Lansing Mi, Michigan State University, East Lansing, MI 48824 USA; Department of Physiology, College of Natural Science, Michigan State University, East Lansing, MI 48824 USA; School of Biological and Population Health Sciences, College of Public Health and Human Sciences, Oregon State University, Corvallis, OR USA

**Keywords:** Primary brain neoplasm, Ketogenic diet, Treatment, Humans

## Abstract

**Background:**

Based on the hypothesis that cancer cells may not be able to metabolize ketones as efficiently as normal brain cells, the ketogenic diet (KD) has been proposed as a complementary or alternative therapy for treatment of malignant gliomas.

**Case presentation:**

We report here our experience in treating two glioma patients with an IRB-approved energy-restricted ketogenic diet (ERKD) protocol as monotherapy and review the literature on KD therapy for human glioma patients. An ERKD protocol was used in this pilot clinical study. In addition to the two patients who enrolled in this study, we also reviewed findings from 30 other patients, including 5 patients from case reports, 19 patients from a clinical trial reported by Rieger and 6 patients described by Champ. A total of 32 glioma patients have been treated using several different KD protocols as adjunctive/complementary therapy. The two patients who enrolled in our ERKD pilot study were monitored with twice daily measurements of blood glucose and ketones and daily weights. However, both patients showed tumor progression while on the ERKD therapy. Immunohistochemistry reactions showed that their tumors had tissue expression of at least one of the two critical mitochondrial ketolytic enzymes (succinyl CoA: 3-oxoacid CoA transferase, beta-3-hydroxybutyrate dehydrogenase 1). The other 30 glioma patients in the literature were treated with several different KD protocols with varying responses. Prolonged remissions ranging from more than 5 years to 4 months were reported in the case reports. Only one of these patients was treated using KD as monotherapy. The best responses reported in the more recent patient series were stable disease for approximately 6 weeks. No major side effects due to KD have been reported in any of these patients.

**Conclusions:**

We conclude that 1. KD is safe and without major side effects; 2. ketosis can be induced using customary foods; 3. treatment with KD may be effective in controlling the progression of some gliomas; and 4. further studies are needed to determine factors that influence the effectiveness of KD, whether as a monotherapy, or as adjunctive or supplemental therapy in treating glioma patients.

**Trial registration:**

ClinicalTrials.gov# NCT01535911

## Background

In 2013, the estimated incidence of primary brain cancers in the United States was 23,130, with 14,080 people dying from this malignancy [1]. Current treatment of primary brain cancers utilizes a multidisciplinary coordinated approach usually involving neurosurgery, radiation therapy, and chemotherapy [2,3]. The median survival period for the most aggressive primary brain malignancy, glioblastoma multiforme (GBM) remains dismal, ranging from 8 to 15 months [4,5]. Combining radiation therapy with temozolomide increases median survival by just 2.5 months [4]. This poor response to current treatments with its associated limited prognosis is the driving force for new and novel therapeutic approaches.

It has been proposed that energy-restricted ketogenic diets (ERKD) might serve as a metabolic treatment to improve survival of primary brain cancer patients [6-9]. The rationale underlying this therapy is based upon the differences between the ability of normal brain cells and tumors to utilize ketones as a metabolic fuel [10,11]. Under normal physiologic conditions, brain cells can obtain energy from either glucose or ketones. In contrast, many tumors become more dependent on glucose for energy support because they have decreased expression of critical ketolytic enzymes [12,13]. In theory, ERKD is predicted to improve survival of GBM patients simply by restricting tumors of glucose, while providing necessary metabolic fuel in the form of ketones to support vital organs including the brain. It is noteworthy that hyperglycemia is associated with adverse prognosis and post-operative function loss in patients with glioblastoma [14,15].

**Figure 1:**
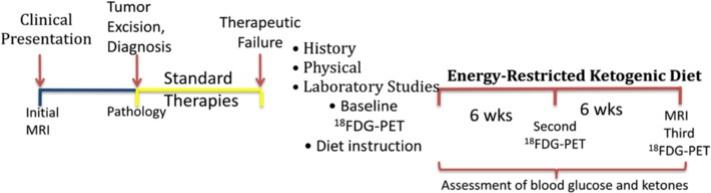
**Schematic overview of the ERKD protocol.** After standard therapies, patients are evaluated with history and physical examination and a PET/CT scan of the brain. These evaluations are repeated after 6 and 12 weeks of diet treatment. After 12 weeks of ERKD therapy, the protocol is completed.

Detailed reports describing the treatment of advanced primary brain malignancies with ERKD have been limited to just five patients [13,16-19]. One recent retrospective study reported the outcomes from six patients who implemented a ketogenic diet as adjunctive or complementary therapy with chemo radiotherapy and adjuvant chemotherapy [6]. An additional 19 patients treated with a registered clinical trial using a ketogenic protocol were recently reported [20]. These limited reports suggest that the ERKD may have antitumor activity in aggressive primary brain cancers. To address the question of whether ERKD as a single modality can either halt disease progression or decrease the size of the tumor mass and improve the patients’ health, we have developed an ERKD protocol (Figure 1). This report reviews the published clinical literature describing the use of a ketogenic diet for the treatment of primary aggressive brain cancer and includes 2 patients treated with the protocol described herein, 5 patients from detailed case reports [13,16-19], 19 patients treated in Germany [20], and 6 patients recently described by Champ [6].

## Case reports

### Methods

Protocol for the energy-restricted ketogenic diet was approved by Michigan State University’s IRB and registered with the NIH at Clinical Trials.gov#NCT01535911.

#### Inclusion criteria:

1. Adult subjects over age 18 with biopsy proven GBM or (WHO grade IV anaplastic astrocytoma);
2. Measureable disease after standard therapies;
3. Immunohistochemical evaluation for the expression of two ketolytic mitochondrial enzymes, succinyl CoA: 3-oxoacid CoA transferase (OXCT-1), and β-3-hydroxybutyrate dehydrogenase 1 (BDH-1), in the patient’s tumor specimen;
4. Eastern Cancer Oncology Group performance status ≤2;
5. Life expectancy >3 months.

#### Exclusion criteria:

1. Diagnosis of diabetes mellitus that is being treated by medication;
2. Concomitant use of glucocorticosteroids;
3. Cholecystectomy within 1 year prior to study entry;
4. Symptoms requiring immediate surgical intervention or radiation therapy;
5. Inability to adhere to or tolerate the dietary protocol;
6. Active malignancy other than primary brain tumor requiring therapy;
7. Participation in an investigational study within 2 weeks prior to study entry;
8. Major co-morbidities such as liver, kidney, or heart failure that in the judgment of the investigators would disqualify the subject from the trial;
9. Pregnancy;
10. Inability to give informed consent.

### Research design

After signing informed consent, the patients were admitted as inpatients to Sparrow Hospital, Lansing, MI. This allowed for initiation of the ERKD, the monitoring of the patients for any side effects from ketonemia and low blood sugar, and education focused on the diet and procedures for monitoring blood glucose and ketones. Initiation of ketonemia was accomplished by an initial supervised fast of approximately 48 h. The subjects were trained by an experienced registered dietitian (RD) to assure competency for adherence to the ERKD protocol. The ERKD protocol was to be administered for 12 weeks as medically appropriate. The diet protocol ideally was to consist of a commercial formula (Ketocal®; Nutricia North America, Gaithersburg, MD) to provide a 3:1 ratio of fat grams to the grams supplied by protein and carbohydrate. Protein adequacy was to be met by ensuring that subjects consumed approximately 0.6 grams protein per kilogram (kg) body weight. Total kilocalories (Kcal) for each subject was estimated based on the ‘ideal body mass index (BMI)’ method (a BMI of 20 to 24.9 formed the basis of this estimate). Estimated caloric intake of each subject is expected to be 20 to 25 Kcal per kg body weight per day with an estimated 20% restriction of calories per day. Training of the subjects to ensure adequate hydration was carried out during the inpatient phase of the study. The subjects were assigned to one of the RDs experienced with administration of ERKD therapy working on the study team. The RD was responsible for follow-up with the subject by phone twice each week to assist with adherence to the ERKD protocol. The subjects were referred to study physicians as necessary to manage any condition or symptom requiring medical care.

### Daily biochemical indices of treatment compliance

The patients and their spouses were trained to utilize the Precision Xtra® Meter made by Abbott Diabetes Care (Alameda, CA). This meter measures blood glucose and ketones (as β**-**3-hyroxybutyrate) simultaneously. Adherence to the diet protocol was monitored by at least twice daily measurement of blood glucose and ketones during the AM fasting period before breakfast and at night 2 h after the evening meal. The subjects were trained to the goal of maintaining blood glucose between 50 and 70 mg/dl and blood ketones between 3 and 8 millimoles per liter (mM). Daily body weights were measured with a body weight scale provided by the study investigators.

### Clinical and laboratory evaluation

The subjects were evaluated with history and physical examinations as well as blood studies including complete blood count, chemical profile, lipids, and uric acid at three time points at the beginning of the study and after 6 and 12 weeks. Because of clinical and image evidence of tumor progression, patient no. 1 discontinued the clinical trial after the fourth week of the ERKD protocol without completing the planned evaluations at 6 and 12 weeks of ERKD. Patient no. 2 did complete the entire 12 weeks of the ERKD protocol.

### Evaluation of patients’ tumor response to ERKD

Patient’s tumor response was evaluated according to the Response Evaluation Criteria in Solid Tumors (RECIST) criteria [21].

### ERKD protocol patients

In order to evaluate the ERKD as monotherapy for advanced, aggressive, primary brain cancers, we developed a 12-week protocol with clinical, laboratory, and imaging evaluations performed immediately before initiating the study and at 6 and 12 weeks after starting the ERKD (Figure 1). The first two patients after signing informed consent were treated with this protocol and are described below.

**Figure 2:**
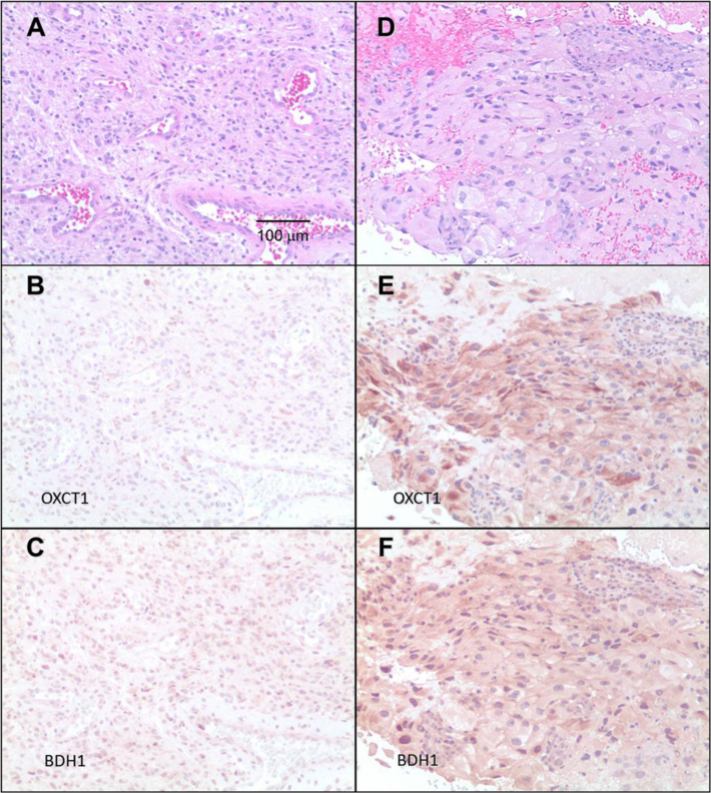
**Immunohistochemistry staining for ketolytic enzymes BDH-1 and OXCT-1. (A-C)** Micrographs of patient no. 1’s tumor. **(A)** H&E stained section. **(B)** Immunohistochemistry reaction shows that most cells in this region, probably tumor cells, demonstrate decreased or ‘low’ expression of OXCT-1. **(C)** Many cells in this same region appear positive for BDH-1. **(D-F)** Micrographs of patient no. 2’s tumor. **(D)** H&E stained section. **(E)** Immunohistochemistry reaction shows that most tumor cells in this region are positive for OXCT-1. **(F)** Most tumor cells in the same region also appear positive for BDH-1. All micrographs were taken at the same magnification (×200).

Patient no. 1, a 55-year-old white male, presented with complete left-sided visual blindness, decreased analytical mental skills, a slow methodical wide-based gait, and a right posterior brain mass with a histological diagnosis of GBM. Twenty months after initial diagnosis and treatment with surgery, radiation therapy, and temozolomide, the patient had documented tumor progression. After signing informed consent, he was treated with ERKD. Patient no. 1’s tumor was evaluated using immunohistochemistry for expression of two mitochondrial ketolytic enzymes: succinyl CoA: 3-oxoacid CoA transferase (OXCT-1) and β-3-hydroxybutyrate dehydrogenase 1 (BDH-1). Expression of these enzymes within the same specimen varied, some regions were scored as decreased, and some were positive (Figure 2).

**Figure 3:**
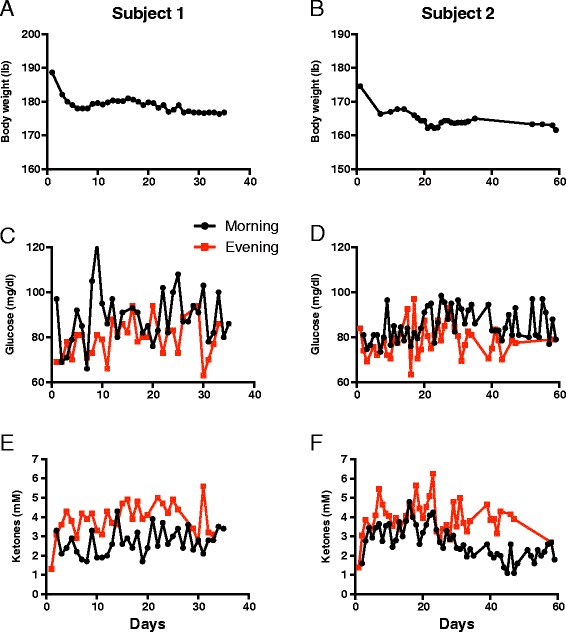
**Blood glucose, ketones, and daily weights.** Twice daily body weights **(A, B)**, blood glucose **(C, D)** and ketones **(E, F)** are graphed for each day the patient was treated with ERKD. Data for patient no. 1 is depicted in the panels **(A, C, E)** to the left and patient no. 2 in the panels **(B, D, F)** to the right.

**Figure 4:**
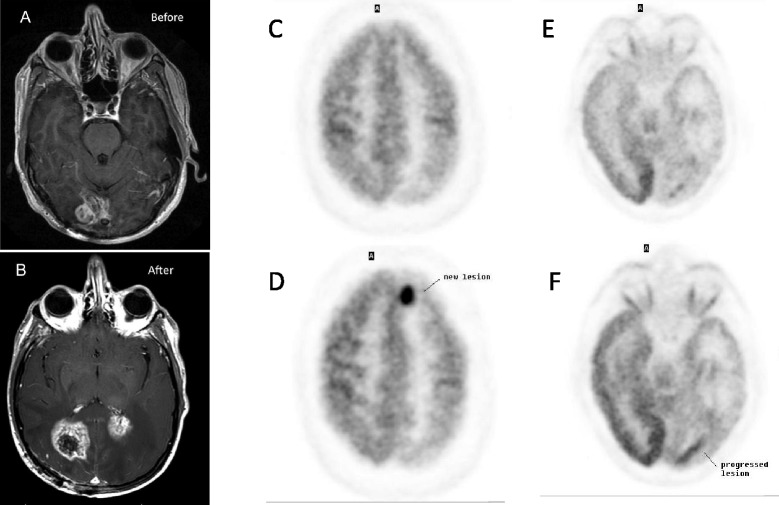
**MRI and PET imaging studies. (A,** **B)** MRI post-contrast T1-weighted images of patient no. 1 showing a right occipital single mass before ERKD **(A)** and a second left occipital mass after ERKD **(B)**. **(C-F)** FDG PET brain images of patient no. 2: **(C)** and **(E)** were done at the beginning of the ERKD protocol. **(D)** and **(F)** are images taken 12 weeks after the initiation of ERKD protocol. These show an area of new disease in the left frontal lobe **(D)** and an area of progressed disease in the left occipital lobe **(F)**.

He was hospitalized to induce a decrease in glucose and an increase in ketones using Ketocal® (Nutricia North America, Gaithersburg, MD) to achieve 20 to 25 Kcal/kg body weight. Diet adherence and efficacy were monitored using AM and PM measurements of blood glucose and ketones. After an initial weight loss of 6% (188 to 176 lbs), the patient’s weight stabilized (Figure 3). Initial treatment with Ketocal® decreased his blood glucose, so that his AM and PM glucoses were <80 mg/dl and increased his AM and PM ketones to >3 mM, meeting the target concentrations stipulated in our study protocol (Figure 3). Because of the low palatability of the Ketocal® formula, the patient was switched 6 days after beginning the ERKD protocol to a ketogenic regular food diet pattern with a 3:1 ratio of fat to combined grams from proteins and carbohydrates. After hospital discharge on this diet, his PM ketones remained >3 mM and his AM >2 mM (Figure 3), but his AM and PM blood glucose increased to >80 mg/dl most of the time (61% of time) (Figure 3). After 4 weeks of treatment with the ERKD, patient 1 withdrew from the study due to further impairment of vision, mobility and cognition, and magnetic resonance imaging (MRI)-demonstrated tumor growth (Figure 4A,B).

Patient no. 2, a 52-year-old white male, presented with a seizure, complete right-sided visual blindness, decreased analytical mental skills, and a left posterior brain mass with a histological diagnosis of GBM. Fourteen months after initial diagnosis and treatment with surgery, radiation therapy, and temozolomide, the patient had documented tumor progression. After signing informed consent, he was treated with ERKD. Initial hospitalization induced ketosis, and during the 12-week study, he remained ketotic. His blood glucose levels could not be maintained below the target value of 80 mg/dl but with a few exceptions remained below 100 mg/dl (Figure 3). Six weeks after starting the diet both his clinical exam and his PET scan demonstrated stable disease. After 12 weeks of ERKD, he had both clinical and radiological evidence of tumor progression. He reported that his vision at times had decreased to the point that he appeared to be seeing through a tunnel. This symptom would wax and wane and usually improved with sleep. In addition, his word-finding skills had decreased. Neurological exam showed an increase in his deep tendon reflexes to 4+ bilaterally accompanied by ankle clonus. Both repeat MRIs and PET scans demonstrated a new medial left frontal mass and an increase in midline deviation to the right from 5 to 13 mm. Blood studies showed that the serum cholesterol increased from 206 at initiation to 281 at 6 weeks and 252 after 12 weeks. His LDL cholesterol also increased from an initial value of 145 to 197 after 6 weeks and 182 at 12 weeks. The patient and his wife reported no significant adverse effects from treatment with the ERKD, except for headaches that occurred between weeks 6 and 8 that were relieved by rest and over the counter headache medication.

Both of these patients (no. 1 and no. 2) who were treated with an ERKD protocol (Figure 1) had progression of their disease. Patient no. 1 progressed after 4 weeks of treatment with the ERKD, and patient no. 2 had stable disease after 6 weeks but had progressed after 12 weeks of diet therapy. One explanation for the failure of the ERKD to control these patients’ tumor progression may be the failure to keep the patient’s glucose in the target range of 50 to 70 mg/dl. Other possibilities include heterogenous expression of the mitochondrial ketolytic enzymes in their tumors. Immunohistochemical evaluation of mitochondrial ketolytic enzymes showed diminished expression of BDH-1 and OXCT-1 in patient no. 1’s original tumor. However, evaluation of his subsequent biopsy showed positive expression of BDH-1 (Figure 2C). The tumor of patient no. 2 was positive for both BDH-1 and OXCT-1 (Figure 2E,F). These data suggest that at least some of the malignant cells in these patients’ cancers could metabolize ketones and derive energy for subsequent growth.

Our experience suggests that it is critical to have at least weekly contact with a knowledgeable registered dietitian, so that the diet can be altered when necessary to maintain target blood levels of glucose and ketones and to have responsible family caregivers assist in all aspects of the diet and blood monitoring. Aside from the inconvenience of altering a patients’ customary diet, side effects attributable to the ERKD were minimal.

### Review of five previously published patient reports

Five patients with advanced brain tumors and favorable responses to ERKD have been reported (patients no. 3 to 7) (Table 1). The best response was a 3-year-old girl who remained in complete remission 5 years after treatment with a ERKD [16]. In four of the five patients, ERKD was combined with one of the standard modalities of treatment, either radiation or chemotherapy. The most recent report showed that three of the five patients were in complete remission and two of the five had documented disease progression after stopping the ERKD.

**Table 1:** **Before ERKD clinical summary**

**Pt. no.**	**Age**	**Sex**	**Pathological diagnosis**	**Tumor locations**	**Before treatment**
1	55	M	GBM	Rt. post cerebral cortex	Surgery, Rad-TX TMZ
2	52	M	GBM	Lt. post cerebral cortex	Surgery, Rad-TX TMZ
3 [16]	3	F	Anaplastic astrocytoma stage (IV)	Entire spinal chord	Rad-TX, Chemo-TX
4 [16]	8.5	F	Cerebellar, low-grade astrocytoma Dx, age 6 years, second surgery cerebellar astrocytoma grade III	Cerebellum	Surgery, age 6, removed 95% of cerebellum; second surgery, age 8, Chemo-TX-CDDP
5 [17]	65	F	GBM	Rt. hemisphere multi-centric location (MRI) shift of Lt. midline structures	Surgery
6 [18]	5	M	Juvenile pilocytic astrocytoma	Thalamus hypothalamus	Chemo-TX, surgery
7 [19]	40	M	GBM	Lt. partial cerebral mass	Surgery, gliadel wafers, Rad-TX, TMZ, Avastin

GBM, glioblastoma multiforme; RT, radiation therapy; TMZ, temozomide; MCT, medium chain triglyceride; CDDP, cisplantin; Rad-Tx, radiation therapy; Chemo-TX, chemotherapy.

The inclusion of the detailed descriptions from these five previously reported cases and the two cases described herein enables a summary discussion of seven individually described patients (four males and three females) with advanced primary brain cancers treated with a ketogenic diet. The salient features of these patients are summarized in Tables 1, 2, 3, and 4. Four of the treated patients were adults, mean age 53 and three were children, mean age 5.5 years. The pathological diagnoses in four patients were GBM, with spinal cord anaplastic astrocytoma and cerebellar astrocytoma grade III, and juvenile pilocytic astrocytoma the diagnoses in the other three patients. The tumors were located in different loci throughout the brain or spinal cord. Before treatment with ERKD, all seven patients were treated with at least two or three different treatment modalities including surgery, radiation therapy, and chemotherapy (Table 1).

**Table 2:** **Imaging and neurological findings**

	**Imaging**		**Neurological**	
**Pt no.**	**Pre ERKD**	**Post ERKD**	**Pre ERKD**	**Post ERKD**
1	Rt. post cerebral mass	Extension of Rt. post cerebral mass	Lt. sided visual field defect, wide based gait, ↓analytical mental skills	Lt. sided visual field defect, difficulty walking, unable to work as engineer, ↑blindness, dementia
2	Lt. post cerebral mass	New mass Lt frontal medial lobe	Rt. visual field defect, ↓analytical and administrative skills	Rt. visual field defect, intermittent tunnel vision, ↓analytical and administrative skills
3	Extensive involvement entire spinal chord	No change in MRI scan. FDG uptake decreased by 21%	↓Body wt, failure to thrive, ↓motor skills	↑Skill development, gait, mobility, speech, hand coordination, could stand and sit and walk with walker
4	Stable cerebellar tumor by CT	FDG uptake ↓21%	↑Headaches, ↓balance, ↓coordination	Unknown
5	Multi-centric: Rt. temporal pole, frontal operculum, insular lobe, post putamen	MRI negative, Pet negative	Progressive memory loss, headaches, ↓vision, Lt. sided facial and arm weakness	Karnoski 100% No neuro deficits
6	MRI thalamic and hypothalamic mass	15% ↓tumor by MRI	↓Vision, hypothalamic obesity, ↓stamina, ↓pituitary function	↑Vision, ↓hypothalamic obesity, ↑stamina, ↑pituitary function
7	Lt. parietal enhancing mass	CT-PET, tumor necrosis	↓Word finding, ↑confusion, blurred vision	Continued working and exercising

FDG, floro-deoxy-glucose.

**Table 3:** **BMI, diet, and ketones**

**BMI**			**Diet**	**Blood**			**Urine**
**Pt. no.**	**Pre ERKD**	**Post ERKD**	**Food and/or Ketocal**	**Estimated total calories/day**	**Glucose (mg/dL)**	**Ketones (mm)**	**Ketones**
1	27	25.4	Initial Ketocal changed to food 3:1 ratio Fat:protein and CHO	20 to 25 Kcal/kg	initially <80 then >80	2 to 4	
2	24.3	22.7	Food 3:1 ratio Fat:protein and CHO	20 to 25 Kcal/kg	Usually <100	2 to 4	
3	17.6	17.6	70 to 85 Kcal/kg 4 to 5 TSP MCT oil				
4	14.6	14.6	11.5 TSP MCT	2,200 Kcal/day	72-90	2 to 4	
5	25.6	20 Good health 100% Karnofsky score	600 Kcal/day Ketocal 4:1 10 gm MCT	600 Kcal/day	<60	1 to 2.5	3+
6	21	18	Atkins ↓ Classic Ketogenic diet 3.5:1 Fat:protein and CHO	80 to 90% est. energy needed	Atkins 77.2 Keto-diet 60 to 85		
7			ERKD		55 to 70	4	

CHO, carbohydrates; MCT, medium chain triglycerides; Prot, protein; TSP tea spoons.

**Table 4:** **Treatment response**

**ERKD treatment response**				**Post ERKD treatment**	
**Pt. no.**	**ERKD monotherapy or multimodality treatment**	**CR, PR, Stable**	**Duration of response**	**Modality**	**Response**
1	ERKD Mono-TX	Progression		Avastin	Clinical neurological deterioration, ↑blindness
2	ERKD Mono-TX	Stable at 6 weeks; progression at 12 weeks	6 weeks	Decadron	
3	Mono-TX	Stable	5 years remission	None	Remission 5 years. Good quality of life
4	Multi modal with Chemo-TX	Stable ?	4 years remission	Chemo-TX	4 years remission good quality of life
5	Multi modal Rad-TX and TMZ	CR	4 months	CPT-11 Bevacizumab	
6	ERKD and Vinblastine	Stable	>12 months		
7	ERKD and Avastin	Stable	4 months	Decadron	

Table 2 summarizes the pre and post ERKD findings for the patients’ imaging and clinical neurological changes. Following ERKD treatment, patients 3, 4, and 6 had no evidence of disease with imaging and/or clinical neurological examination at the time of the report, but patients 1, 2, 5, and 7 had documented evidence of tumor growth.

The metabolic changes associated with ERKD and the different approaches to implementing an ERKD for each patient are summarized in Table 3. Body mass index, reported in six patients, did not decrease more than 20%. Patient 5 was treated part of the time with 600 Kcal/day, and her BMI decreased from 25 to 20 (20%). Initially, patient no. 1 was treated with Ketocal® which maintained his glucose and ketones within the desired range. Because of the poor palatability of the Ketocal®, he elected to be changed to an ERKD using ketogenic food pattern. This change in type of ketogenic diet modality resulted in his blood glucose increasing above the target range for our study while his serum ketones remained above 2 mm. Patients 3, 4, and 5 used medium chain triglycerides (MCT; as MCT oil) as a source of fat and patient no. 6 was initially started on a classical Atkins Diet and changed to an ERKD with a ratio of 3.5:1 grams of fat to combined grams of protein and carbohydrates. Most of the patients were able to keep their serum ketone levels above 2 or 3 mm. However, a target serum glucose ranging between 50 and 70 mg/dl was not always achieved and did not appear to be absolutely required in patients 3, 4, and 5 who achieved long-term disease free survival. Perhaps, the inability to decrease blood glucose into the target range was because of standard of care treatments and/or because the prescribed calorie decrease was limited. In the two new patients reported, the prescribed calorie restriction was limited to 20%.

The duration of response of the five previously published patients ranged from 4 months to more than five years (Table 4). Four patients were simultaneously treated with another treatment modality such as radiation therapy and/or chemotherapy in addition to treatment with the ERKD. When the patients relapsed, they were treated with chemotherapy that may have included bevacizumab and/or decadron. One of the 3 patients treated with ERKD as monotherapy responded and was alive with no evidence of tumor progression 5 years later [16].

All of the five previously reported patients (patients no. 3 to 7) who were reported in detail responded to the ERKD. Maintenance of blood glucose at <60 mg/dl was only accomplished in patient no. 5 who limited her daily calorie intake to 600 Kcal/day. Serum ketones reported in four patients varied from 1 to 4 mm. Serial PET scans obtained on two patients showed a 21% decrease in tumor glucose uptake during treatment with ERKD. Because these patients were treated without a common treatment protocol and without periodic clinical, laboratory, and imaging evaluations, efficacy of the ERKD as single modality therapy cannot be assessed.

### Ketogenic diet treatment of patients, two clinical studies

Rieger and co-workers reported their results of a pilot study in Germany treating recurrent glioblastoma with a ketogenic diet (KD) [20]. They observed no serious adverse effects directly related to the diet and showed that the study was feasible which was their primary endpoint. Their study was not designed to test antitumor effects of the KD. Prescribed measurements of blood glucose and ketones were not performed. Patients tested their urine for ketones and 12 of the 13 evaluable patients had at least one urine positive for ketones. The patient’s calorie intake was not restricted; they were instructed to eat to satiety. The diet was not supervised by a registered dietitian. Patients were given a set of brochures with sample cooking recipes and food facts. In addition, patients were treated with steroids; eight patients were treated with dexamethasone before the diet and 11 patients received this drug during the diet treatment. Of the 19 patients, three discontinued the treatment because of poor tolerability. Of the 16 remaining patients, two had stable disease and one had a minor response. A trend towards an increase in progression-free survival was reported in patients with stable ketosis.

A retrospective review of 53 patients with high-grade glioma treated with concurrent chemo radiotherapy and adjuvant chemotherapy was carried out to determine the association between ketogenic diet, adherence and survival, serum glucose and ketone levels, and dexamethasone dose [6]. Blood glucose levels were compared between patients on an unspecified/standard diet and a KD. Of the 53 patients, six underwent a KD during treatment. The non-standardized Atkins/low carbohydrate diets were well tolerated with no documented symptomatic hypoglycemic episodes or grade III toxicity. One episode of grade II fatigue was reported. Four of six patients were alive at a median follow-up of 14 months. At the time of the report, two of the four living patients had recurrence and one was without evidence of disease 12 months after starting treatment. Investigators reported that the mean blood glucose of patients on a regular, non-controlled diet was 122 versus 84 mg/dl for those reporting adherence to a KD. Based on this retrospective study, a KD appears safe and well tolerated during the standard treatment of GBM. It was noted that the retrospective design did not allow for determination of whether dietary restriction of carbohydrates or KD adherence was associated with the observed reduction in serum glucose levels. It is significant that reductions in blood glucose were observed with KD adherence even in conjunction with high dose steroid treatment.

## Discussion

This review of the two patients treated by the described protocol, the previous detailed case reports, and the case series reported by Rieger and by Champ suggests that ERKD has minimal side effects and may be helpful in controlling some primary brain cancers. These studies serve to generate critical questions that, if addressed in rigorous clinical protocol studies, may have application to patient care.

Some of the critical questions are as follows:

1. Is an ERKD effective as a single modality treatment in patients with aggressive brain cancer?
2. If the ERKD is effective in some patients, can outcomes be enhanced by limiting treatment to patients with decreased expression of the mitochondrial ketolytic enzymes BDH-1 and OXCT-1, or other metabolic enzymes?
3. What is the optimal diet and calorie consumption per day that will maximize the antitumor effect?
4. What range of blood concentrations for increased serum ketones and decreased blood glucose are associated with maximal antitumor effect?

## Conclusions

Studies to answer these questions require a common protocol, with individual patient supervision by an experienced dietitian to adjust the patient’s diet after evaluating twice daily measurements of blood glucose and ketones and daily weights. Only with this kind of clinical study will the efficacy of the ERKD in treatment of aggressive primary brain cancers be adequately evaluated. The use of the ERKD as an adjunctive therapy for GBM is promising; assessing the clinical utility of this therapy in larger prospective studies is dependent upon consensus regarding safety, validation of potential biomarkers of efficacy, and a standardized protocol for patient diet monitoring and evaluation.

## Abbreviations

GBM: glioblastoma multiforme
ERKD: energy-restricted ketogenic diets
OXCT-1: succinyl CoA: 3-oxoacid CoA transferase
BDH-1: β-3-hydroxybutyrate dehydrogenase 1
Kcal: kilocalories
BMI: body mass index
RD: registered dietitian
kg: kilogram
MCT: medium chain triglycerides
KD: ketogenic diet
FDG: flouro-deoxy-glucose
MRI: magnetic resonance imaging
PET: positive emission tomography
CAT: computerized axial tomography
